# Heterologous expression of cyclodextrin glycosyltransferase from *Paenibacillus macerans* in *Escherichia coli* and its application in 2-O-α-D-glucopyranosyl-L-ascorbic acid production

**DOI:** 10.1186/s12896-018-0463-9

**Published:** 2018-08-31

**Authors:** Yujia Jiang, Jie Zhou, Ruofan Wu, Fengxue Xin, Wenming Zhang, Yan Fang, Jiangfeng Ma, Weiliang Dong, Min Jiang

**Affiliations:** 1 State Key Laboratory of Materials-Oriented Chemical Engineering, College of Biotechnology and Pharmaceutical Engineering, Nanjing Tech University, Puzhu South Road 30#, Nanjing, 211800 People’s Republic of China; 2 Jiangsu National Synergetic Innovation Center for Advanced Materials (SICAM), Nanjing Tech University, Nanjing, 211800 People’s Republic of China

**Keywords:** Cyclodextrin glucanotransferase, Optimized codons, Glycosyl donors, 2-O-α-D-glucopyranosyl-L-ascorbic acid

## Abstract

**Background:**

Cyclodextrin glucanotransferase (CGTase) can transform L-ascorbic acid (L-AA, vitamin C) to 2-O-α-D-glucopyranosyl-L-ascorbic acid (AA-2G), which shows diverse applications in food, cosmetic and pharmaceutical industries.

**Results:**

In this study, the *cgt* gene encoding α-CGTase from *Paenibacillus macerans* was codon-optimized (*opt-cgt*) and cloned into pET-28a (+) for intracellular expression in *E. coli* BL21 (DE3). The Opt-CGT was purified by Ni^2+^-NTA resin with a 55% recovery, and specific activity was increased significantly from 1.17 to 190.75 U·mg^− 1^. In addition, the enzyme was adopted to transform L-AA into 9.1 g/L of AA-2G. Finally, more economic substrates, including β-cyclodextrin, soluble starch, corn starch and cassava starch could also be used as glycosyl donors, and 4.9, 3.5, 1.3 and 1.5 g/L of AA-2G were obtained, respectively.

**Conclusions:**

N-terminal amino acid is critical to the activity of CGTase suggested by its truncation study. Furthermore, when the Opt-CGT was flanked by His_6_-tags on the C- and N-terminal, the recovery of purification by Ni^2+^-NTA resin is appreciably enhanced. α-cyclodextrin was the ideal glycosyl donor for AA-2G production. In addition, the selection of low cost glycosyl donors would make the process of AA-2G production more economically competitive.

## Background

Ascorbic acid (Vitamin C, L-AA, Vc) is an essential vitamin in maintaining the health of body, which could not be synthesized naturally [1]. However, its hydroxyl group in the position of C-2 is extremely instable, especially in oxidative, lighted and hot environment, so the application of L-AA is subjected to a lot of restrictions [2]. 2-O-α-D-glucopyranosyl-L-ascorbic acid (AA-2G) is a type of glucosylated L-AA, which is widely used in cosmetic, health products or other fields. In addition, it can play the role in whitening the skin owing to the L-AA released by α-glucosidase from AA-2G. Five kinds of enzymes could catalyze the AA-2G production, including α-amylase, α-glucosidase, sucrose phosphatase, α-isomaltosyl glucosaccharide-forming enzyme and cyclodextrin glycosyltransferase (EC 2.4.1.19, CGTase). Among them, CGTase with higher substrate specificity, higher production and more excellent thermal stability was considered to be the best enzyme for industrial production of AA-2G [3–6].

CGTase is an industrially important enzyme for α-, β- or γ-cyclodextrins (CDs) production, which are extensively used in agriculture, chemicals, cosmetics, foods and pharmaceuticals [7]. Actually, CGTase has been discovered in a great number of *Bacillus*, *Thermoanaerobacter*, *Brevibacterium* and *Thermoanaerobacterium* [8–10]. The CGTase from *Bacillus* genus belonging to glycoside hydrolases family 13 (GH13) is the most commonly used one, which can catalyze three kinds of reactions: cyclization, intramolecular transglycosylation and low hydrolytic reaction [11–13]. In addition, intramolecular transglycosylation reaction includes disproportionation reaction (transfer the donor substrate to acceptor substrate) and coupling reaction (cleave cyclodextrins and transfer the resulting maltooligosaccharide to acceptor substrate) [11].

The productivities of CGTases from wild strains are relatively low, partly resulting in high production costs for industrial applications. Currently, α-CGTases were mainly obtained by extracellular heterologous expression in *E. coli* or *B. subtills*. Compared with extracellular expression, intracellular expression would simplify the preservation, facilitate enzyme enrichment and isolate the disturbance of the external circumstances. In addition, typical yield of purified α-CG Tas was at a relatively low level by one-step affinity chromatography on Ni^2+^-NTA resin, and > 95% of the recombinant enzyme was left in the flow-through fraction because of the C-terminal His_6_-tag was partially inaccessible [14].

Previous reports showed that abundant biologically inactive inclusion bodies of α-CGTases accumulated when it was expressed intracellularly [14, 15]. Codon optimization is known to be an important strategy which could improve the levels of gene soluble expression in the host and ultimately affects enzyme production [16]. To enhance the α-CGTase soluble expression, the α-CGTase encoding gene from *Paenibacillus macerans* JFB05–01 was codon-optimized first, and then expressed in *E. coli* BL21(DE3) intracellularly. The recombinant Opt-CGT was flanked by His_6_-tags on the C- and N-terminal to increase binding affinity with Ni^2+^-NTA resin. The enzymatic properties were characterized and its ability of transforming L-AA to AA-2G was investigated. In addition, other more economic glycosyl donors were selected in order to decrease the cost of AA-2G production.

## Methods

### Chemicals and media

Peptone and yeast extract were purchased from Oxoid Co. Ltd. (Beijing, China), soluble starch was produced by Xilong chemical Co. Ltd. (Guangzhou, China), α-cyclodextrin was purchased from Sangon Biotech. Co. Ltd. (Shanghai, China) and the other chemical reagents were purchased from Lingfeng chemical reagents Co. Ltd. (Shanghai, China). All molecular biology reagents were purchased from TaKaRa Co., Ltd. (Dalian, China). All chemicals used in this study were of analytical grade or a higher purity. The Luria-Bertani (LB) medium was used to culture the *E. coli* strains in this study. The improved terrific broth (ITB) medium containing 15 g/L maltose, 12 g/L peptone, 24 g/L yeast extract, 16.43 g/L K_2_HPO_4_·3H_2_O and 2.31 g/L KH_2_PO_4_ was used for the fermentation of recombinant strain.

### Strain, plasmid and primers

Secondary structure in dot-bracket notation with a minimum free energy (MFE) could be predicted using the ViennaRNA Web Services (http://rna.tbi.univie.ac.at/) [17]. Codon adaptation index (CAI) based on the set of highly expressed genes from *E. coli* was calculated using the CAIcal server (http://genomes.urv.es/CAIcal/) [18]. The mRNA senior structure and possible RNA stem structure were predicted by a web site of http://www.genebee.msu.su/services/rna2_reduced.html. Finally, the α-CGTase encoding gene (*cgt*) from *P. macerans* JFB05–01 was codon-optimized by GeneWiz, Inc. (Suzhou, China) and the resulting gene was named as *opt-cgt*. The expression vector pET28a (+) with two His_6_-tags at both of C-terminal and N-terminal was used. The *E. coli* BL21 (DE3) was used as host strain and routinely grown aerobically at 37 °C in LB broth or on LB agar. All the primers used in the study were listed in Table 1.

**Table 1:** Oligonucleotide primers used for PCR

Primers	Oligonucleotide sequences (5′ to 3′)
F1	5′-CAGCAAATGGGTCGCGGATCCAGTCCTGACACCAGCGTGG-3′
R1	5′-GGTGGTGGTGGTGGTGCTCGAGATTCTGCCAGTCAACGGTCACTG-3′
F2	5′-CAGCAAATGGGTCGCGGATCCCCTGACACCAGCGTGGATAACAAGG-3′
R2	5′-GGTGGTGGTGGTGGTGCTCGAGATTCTGCCAGTCAACGGTCACTGTA-3′
F3	5′-CAGCAAATGGGTCGCGGATCCAGCGTGGATAACAAGGTGAACTTTAGT-3′
R3	5′-GGTGGTGGTGGTGGTGCTCGAGATTCTGCCAGTCAACGGTCA-3′
F4	5′-CAGCAAATGGGTCGCGGATCCGATAACAAGGTGAACTTTAGTACAGAT-3′
R4	5′-GTGGTGGTGGTGGTGCTCGAGATTCTGCCAGTCAACGGT-3′
F5	5′-CAGCAAATGGGTCGCGGATCCGTGAACTTTAGTACAGATGTTATTT-3′
R5	5′-GTGGTGGTGGTGGTGCTCGAGATTCTGCCAGTCAACGGT-3′

Underlined segments represent restriction enzyme cutting sites

### Cloning and expression of the *opt-cgt* gene encoding α-CGTase

The *opt-cgt* gene without signal peptide was amplified using the primers F1/R1 listed in Table 1 using PrimeSTAR HS DNA polymerase. Then, the PCR products were inserted into the *Bam*H I and *Xho* I sites of the DNA pET28a (+) plasmid to produce the pET-*opt-cgt* plasmid by One Step Cloning Kit (Vazyme Biotech. Co., Ltd., Nanjing, China). The recombinant plasmid was transformed into *E. coli* BL21 (DE3) and designated as *E. coli* BL21 (DE3-pET-*opt-cgt*). Finally, the insertion of the genes was confirmed by DNA sequencing (GenScript Biotech. Co, Ltd., Nanjing, China).

*E. coli* BL21 (DE3-pET-*opt-cgt*) was incubated in 5 mL of LB medium supplemented with 30 μg/mL of kanamycin at 37 °C for 12 h as the inoculum. The 1.0 mL inoculum was inoculated into a 500 mL Erlenmeyer flask containing 100 mL ITB medium with 30 μg/mL kanamycin and then the condition was maintain at 200 r·min^− 1^ and 37 °C until the optical density at 600 nm (OD_600_) reached 0.5 to 0.6. Then, protein expression was induced with 0.01 mM IPTG and the culture temperature was lowered to 18 °C for 20 h. The bacterial cells were harvested by centrifugation (Eppendorf Centrifuge 5810 R, F-34-6-38 rotor, Brinkman Instruments Inc., USA) at 8085×g and 4 °C for 10 min, washed twice with 50 mM PBS (pH 6.0). The *E. coli* BL21 (DE3-pET-*opt-cgt*) cells were disrupted by a scientz-II D ultrasonic generator (Ningbo Scientz Biotech. co., Ltd., Ningbo, China) in the ice bath for 10 min. The cell debris was removed by centrifugation at 8085×g and 4 °C for 20 min.

In addition, the effects of Opt-CGT N-terminal amino acids on enzymatic activity were carried out by truncating expression. Four PCR fragments (cut off one amino acid, four amino acids, six amino acids and nine amino acids) were amplified using the primers F2/R2, F3/R3, F4/R4 and F5/R5, respectively (Table 1). Then, the four PCR fragments were inserted into pET28a (+) vector to produce the pET-*cgt*-t1AA, pET-*cgt*-t4AA, pET-*cgt*-t6AA and pET-*cgt*-t9AA plasmids which were transformed into *E. coli* BL21 (DE3) as described above.

### Purification and SDS-PAGE

The recombinant Opt-CGT with His_6_-tags was purified with Ni^2+^-NTA resin (Qiagen, Valencia, CA, USA) [19]. After washing of non-target proteins with 25 mM imidazole in 50 mM PBS (pH 7.0), the target fusion protein was eluted with a linear concentration gradient of imidazole in 50 mM PBS (pH 7.0). The fractions containing target protein were collected and dialyzed against 50 mM PBS (pH 7.0) overnight at 4 °C to remove imidazole. The purified Opt-CGT and truncated proteins were diluted with buffer solution to 100 mL and stored at 4 °C, respectively.

The proteins were electrophoresed by sodium dodecyl sulfatepolyacrylamide gel electrophoresis (SDS-PAGE) and visualized by staining with Coomassie Brilliant Blue R-250 [20, 21]. SDS-PAGE was performed on a 12% gel using electrophoresis apparatus at 80 V for the first 60 min, followed by 120 V for 2 h.

### Assay for cyclization activity of α-CGTase

α-Cyclodextrin activity was determined using the methyl orange method during starch hydrolysis [6]. The 0.1 mL of Opt-CGT was incubated in 0.9 mL of 50 mM PBS (pH 6.0) containing 3% (*w*/*v*) soluble starch at 40 °C for 10 min. Then, 1.0 mL of 1 M HCl was added to terminate the reaction and provided an acidic environment, and 1.0 mL of 0.1 mM methyl orange in 50 mM PBS (pH 6.0) was also added. Then the reaction mixture was incubated at 16 °C for 20 min and the amount of α-cyclodextrin in the mixture was spectrophotometrically determined by measuring the absorbance at 505 nm. The standard curve was calculated according to the OD_505_ of different concentration of pure AA-2G. One unit of α-cyclodextrin-forming activity was defined as the amount of enzyme that was able to produce 1 μmol of α-cyclodextrin per min.

### Biochemical properties of the purified Opt-CGT

#### Effects of temperature and pH on Opt-CGT activity and stability

The optimal reaction pH was assessed at 40 °C using the following buffers: 50 mM citrate buffer, pH 4.0–6.0; 50 mM PBS, pH 6.0–8.0; 50 mM glycine-NaOH buffer, pH 8.0–10.0. The effect of temperature on Opt-CGT activity was determined under the optimal pH at different temperatures ranging from 16 to 80 °C. To measure pH stability, the enzyme was incubated at 4 °C for 1 h in different buffers and the residual activity was determined. The thermal stability of Opt-CGT was assessed by incubating the enzyme preparations at different temperatures for a certain time until the remaining activity decreased below 50% of its initial cyclization activity. Non-heated enzyme was used as the control (100%).

#### Effects of metal ions and chemical agents on Opt-CGT activity

Purified Opt-CGT was treated with 1 mM EDTA for 5 h at 4 °C and then dialyzed against 50 mM PBS (pH 6.0) to remove the EDTA. For reactivation, the metal-free enzyme was incubated with divalent metal ions (Fe^2+^, Ba^2+^, Cu^2+^, Co^2+^, Ni^2+^, Zn^2+^, Ca^2+^, Mg^2+^ and Mn^2+^) at a final concentration of 1 mM for 10 min, followed by the remaining activity determination. The procedure used to assess the metal ions was also used to determine the effects of chemical agents (Triton X-100, SDS, Tween20, Tween80 and urea) and organic reagents (methanol, ethanol, acetonitrile, isopropanol and DMSO) [22, 23]. The activity in the absence of any additives was used as the control (100%).

### Assay for AA-2G synthesis

The original reaction mixture in 50 mM PBS (pH 5.0) contained 100 g/L of L-AA, 100 g/L of α-cyclodextrin and 60 U of Opt-CGT. The reaction mixture was incubated at 40 °C for 24 h in the dark and filled with nitrogen_._ In order to hydrolyze the AA-2-oligosaccharides (AA-2Gs) to AA-2G, the glucoamylase was added to the mixture at a final concentration of 10 U/mL. The reaction mixture was incubated at 55 °C for another 24 h. What’s more, the glycosyl donors were also optimized, and α-cyclodextrin, β-cyclodextrin, soluble starch, corn starch and cassava starch were all used as the glycosyl donors.

A separation column (internal diameter, 4.6 mm; length, 250 mm) filled with Kromasil 100–5-C_18_ was used for AA-2G HPLC analysis. The mobile phase was 25 mM KH_2_PO_4_: methanol (99.5:0.5, vol/vol) and adjusted pH to 2.0 using H_3_PO_4_. The flow rate was 0.8 mL/min and temperature of column was 25 °C. The detection wavelength was 238 nm. The retention time of the pure AA-2G was 13.034 min, and the amount of AA-2G was calculated on the basis of the peak area.

## Results and discussion

### Cloning and expression of the *opt-cgt* gene

The Opt-CGT was successfully expressed in *E. coli* BL21 (DE3) according to the results of SDS-PAGE and the analysis of activity. The activity of CGTase reached 6.25 U/mL after IPTG-induced 20 h. Generally, *P. macerans* should be incubated at 37 °C for 72 h to obtain the CGTase [24]. Hence, the fermentation time was shortened significantly by intracellular heterologous expression in *E. coli* comparing to that for *P. macerans*. In addition, the *cgt* gene was also cloned into pET-20b (+) and extracellularly expressed in *E. coli* with a higher enzyme activity of 22.5 U/mL and longer fermentation time of 90 h [14].

Codon bias was considered as the most important determinant of translation efficiency, expression level and the folding energy of mRNA [25]. The value of CAI increased from 0.74 to 0.86 for *E. coli* after the optimization of *cgt* gene. In theory, the CAI of 1.0 is considered as ideal, while a CAI of more than 0.8 is rated as good for expression in the desired expression organism [26]. The minimum free energy (*ΔG*) of the optimal secondary structure in dot-bracket notation was − 770.9 kcal·mol^− 1^ (1 cal = 4.2 J), while the *ΔG* of non-optimized codons was − 774.10 kcal·mol^− 1^ by ViennaRNA Web Services. Yin et al. reported that *ΔG* is closely related to translation efficiency and increasing *ΔG* can enhance the expression level [27]. Amaral et al. demonstrated that the folding free energy of the 5′ end of mRNA transcripts could have significant effects on translation efficiency and overall protein abundance [18]. Otherwise, the number of stable stem regions which has lower energy under − 15 kcal·mol^− 1^ was decreased from 15 to 10 after codon optimization. The lower energy was advantageous to soluble heterologous expression in *E. coli* [28].

### Optimization of expression conditions and purification of Opt-CGT

**Fig. 1:**
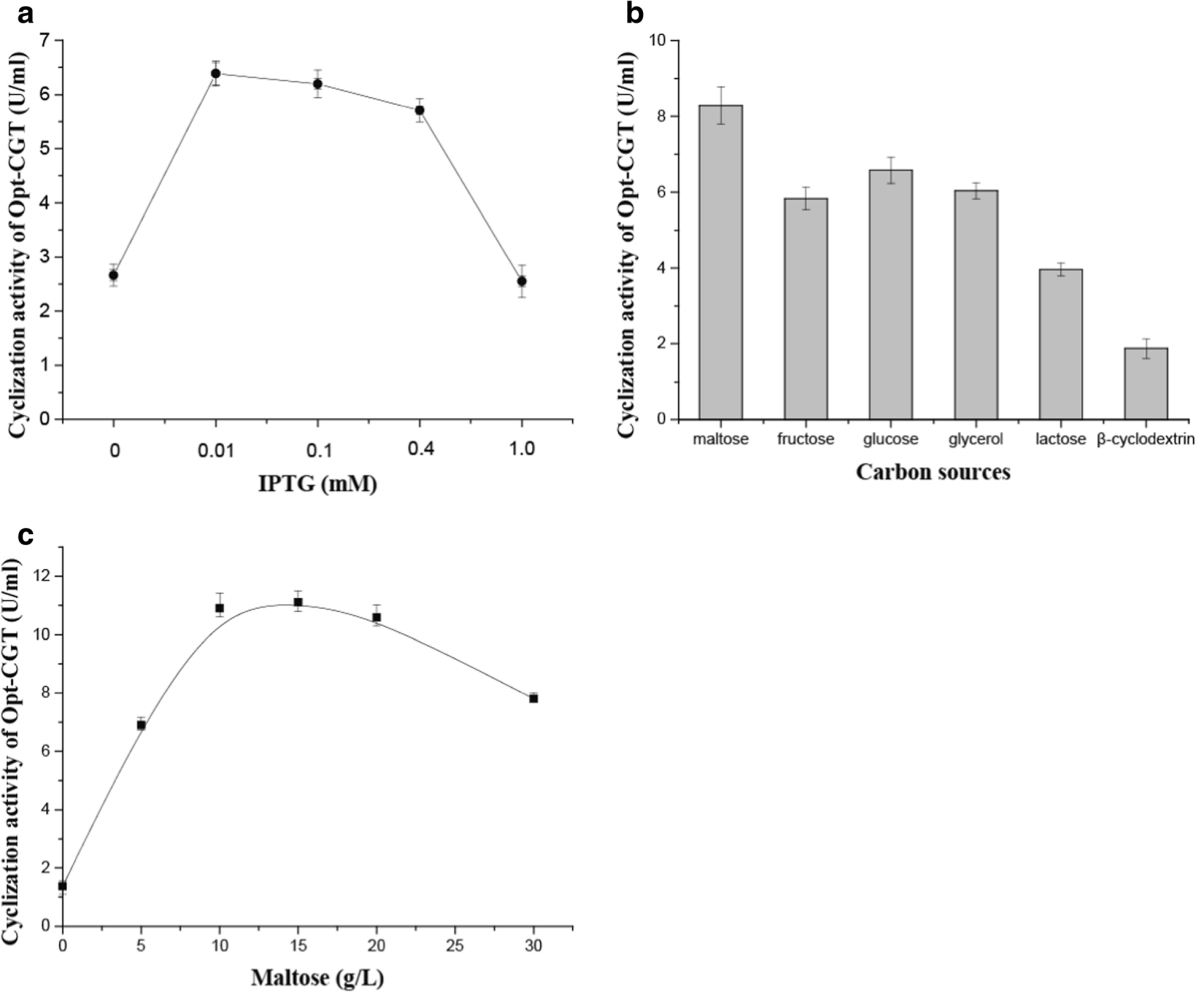
Condition optimization for Opt-CGT expression. **a** effects of different IPTG concentration; **b** effects of different carbon sources; **c** effects of maltose concentration on intracellular expression of Opt-CGT. Error bars correspond to the standard deviation of three measurements

In order to improve the enzyme activity of Opt-CGT, expression conditions were further optimized, including IPTG concentration and carbon sources. The optimized conditions for Opt-CGT expression was 200 r·min^− 1^ and 18 °C for 20 h with 0.01 mM of IPTG. The highest cyclization activity of Opt-CGT reached 11.12 U/mL when 15 g/L of maltose was used as the carbon source (Fig. 1 a, b and c). In general, intracellular expression of α-CGTase is more beneficial to the collection of enzyme. However, the enzyme activity is lower than that of extracellular expression. For example, *α-cgt* was cloned into pET-20b (+) and expressed in *E. coli* with the extracellular enzyme activity of 55.3 U/mL [29].

**Fig. 2:**
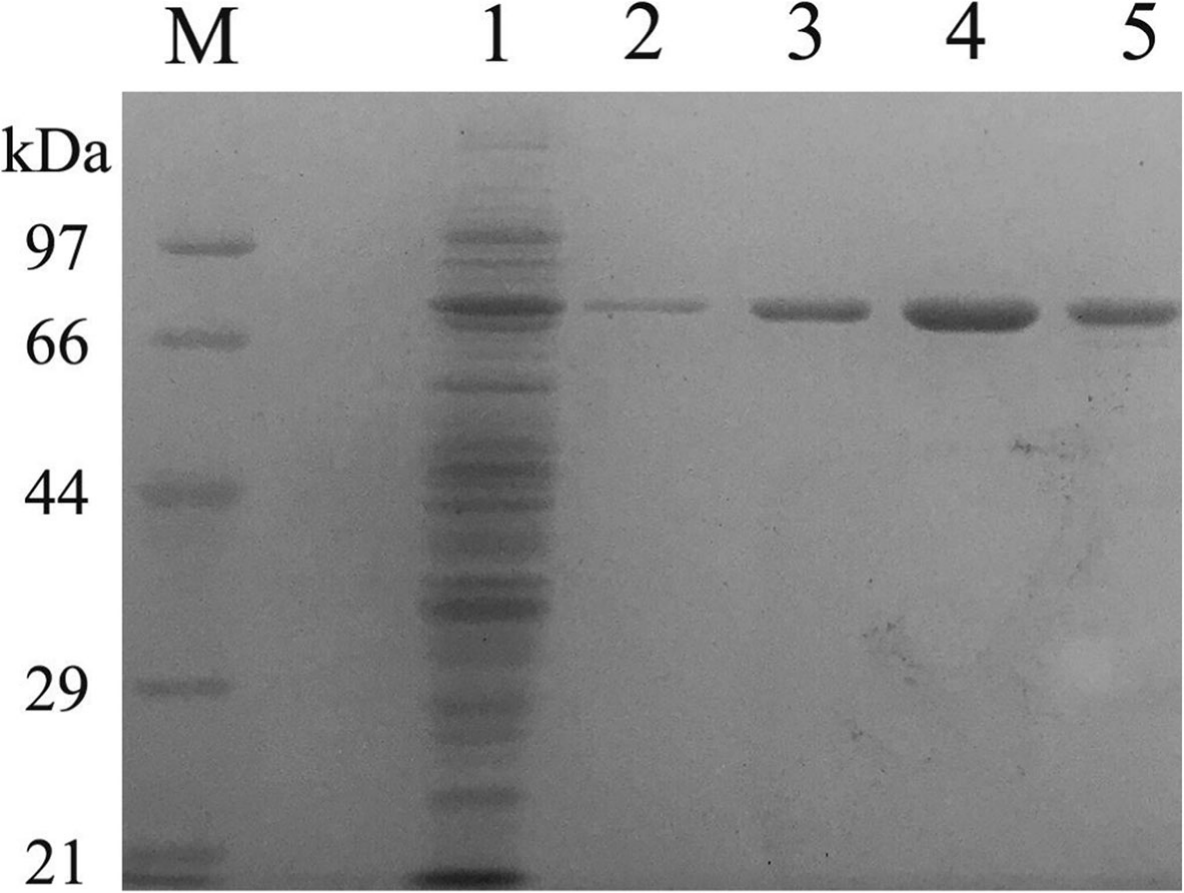
SDS-PAGE analysis of the purified recombinant Opt-CGT. *M* protein marker; *Lane 1* unpurified total soluble proteins from induced BL21 (DE3) harboring pET-*opt-cgt*; *Lane 2–5* soluble proteins purified by nickel affinity chromatography from induced BL21 (DE3) harboring pET-*opt-cgt* (15 μL)

The recombinant Opt-CGT was eluted by Ni^2+^-NTA resin and purified recombinant Opt-CGT was analyzed using SDS-PAGE (12%). The purified enzyme gave a single band on SDS-PAGE (Fig. 2). The molecular mass of the denatured enzyme was approximately 74 kDa, which was in good agreement with the theoretical molecular mass. After the Ni^2+^-NTA resin purification process, Opt-CGT was purified 163-fold with a 55% recovery and specific activity was increased significantly from 1.17 to 190.75 U·mg^− 1^. This is a similar specific activity with the reported two-step purification scheme (Q-sepharose and phenyl-superose), which the specific activity of purified recombinant α-CGTase was 198.82 U·mg^− 1^ [14] (Table 2). However, the recovery yield of purified enzyme reported by Li et al. was less than 5% by one-step affinity chromatography on Ni^2+^-NTA resin, which is a relatively low level. The increased yield of purified enzyme maybe because the plasmid of pET28a (+) flanked by His_6_-tags on the C- and N-terminal, which could increase Opt-CGT binding affinity with Ni^2+^-NTA resin.

**Table 2:** Comparison of purification process between native CGTase and Opt-CGT

Details	Opt-CGT (This study)	Native CGTase
Origin Organism	*Paenibacillus macerans* JFB05–01	*Paenibacillus macerans* JFB05–01
Codon optimization	Yes	No
CAI (*E. coli*)	0.86	0.74
Plasmid/host	pET-28a (+)/*E. coli* BL21	pET-20b (+)/*E. coli* BL21
Induction time (h)	20	90
Total protein of crude enzyme (mg)	330	401
Purification methods	Ni^2+^-NTA	Q-Sepharose + Phenyl-Superose
Total protein of purified enzyme (mg)	7.66	8.31
Specific activity (U/mg)	190.75	198.82
Purification fold	163	13.17
Yield (%)	55	27.27

### Effects of N-terminal amino acid on cyclization activity of Opt-CGT

The NH_2_ domain of CGTase was considered more important for cyclization [8]. In this study, 1 amino acid, 4 amino acids, 6 amino acids and 9 amino acids were truncated in the N-terminal of Opt-CGT, respectively. These truncated Opt-CGTs were designated CGT-t1AA, CGT-t4AA, CGT-t6AA and CGT-t9AA, which were expressed in *E. coli* BL21 (DE3), respectively. SDS-PAGE results indicated that all samples were highly expressed in *E. coli* (data not shown). However, the cyclization activity of CGT-t1AA was almost unchanged, and the activity of CGT-t4AA and CGT-t6AA was significantly reduced, whereas no activity could be detected by truncating 9 amino acids of Opt-CGT.

Salt bridges were important for the structure and function of enzymes, such as in the model metalloprotease PAE [30]. Based on the crystal structure of CGTase, two important salt bridges were found in the first 9 amino acids of CGTase at N-terminal, including Asp3-Arg521 and Lys9-Asp224. Three conserved amino acids (Asp229, Glu258 and Asp329) constituted the catalytic residues, which are important for cyclization activity of α-CGTase [31, 32]. Asp224 is closed to the Asp229 which is the active center of α-CGTase, thus, the destruction of the salt bridge Lys9-Asp224 may be the main reason to make Opt-CGT inactivation. The fact that the activity of CGT-t4AA and CGT-t6AA decreased but not completely lost maybe because Arg521 is relatively far from activity center of α-CGTase. This result could suggest the necessity of the N-terminal amino acids for Opt-CGT.

### Biochemical properties of purified opt-CGT

#### Effects of temperature on opt-CGT activity and stability

**Fig. 3:**
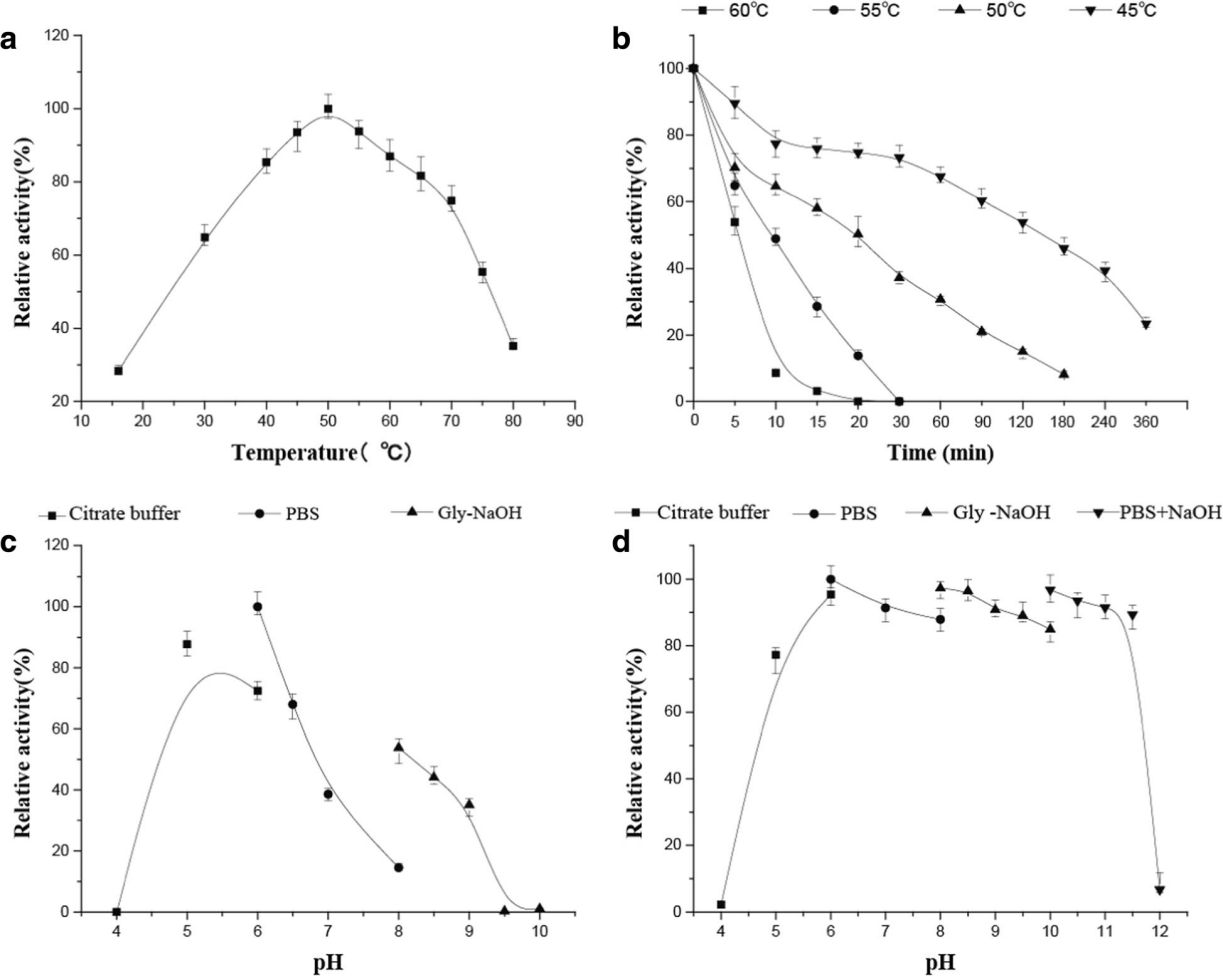
Effects of temperature and pH on enzyme activity and stability of the Opt-CGT. **a** determination of the optimum temperature; **b** thermal stability; **c** determination of the optimum pH; **d** pH stability. Error bars correspond to the standard deviation of three measurements

In this study, the optimum temperature of the recombinant Opt-CGT was 50 °C for the cyclization activity (Fig. 3a). The recombinant enzyme exhibited more than 80% of maximum cyclization activity between 40 °C and 65 °C (Fig. 3a). Opt-CGT was stable and retained > 80% residual activity for 30 min at temperatures < 45 °C, but was unstable at temperatures > 50 °C (Fig. 3b). These results indicated that Opt-CGT was a mesophilic enzyme.

#### Effects of pH on Opt-CGT activity and stability

Opt-CGT exerted high levels of activity at pH 5.0–6.5 with an optimum pH of 6.0 (Fig. 3c). Almost no activity was detected at pH below 4.0 or above 9.5. The recombinant Opt-CGT was more active in glycine-NaOH buffer compared with that in PBS buffer at the same pH. The stabilities of recombinant Opt-CGT for the cyclization activity was also examined in the pH range of 4.0–12.0 (Fig. 3d). Opt-CGT retained > 70% of its activity after storing at pH 5.0–11.5 for 1 h, whereas its stabilities decreased significantly at pHs below pH 5.0 or above pH 11.5 [14].

#### Effects of metal ions and chemical agents on Opt-CGT activity

To determine whether metal ions would affect the cyclization activity, the residual activity of metal-free enzyme was assayed by incubation with 1 mM of divalent metal ions for 10 min. As shown in Tables 3, 1 mM of EDTA almost did not inhibit the enzymatic activity, indicating that the Opt-CGT represented a divalent metal ion non-dependent glycosyl transferase. Zn^2+^, Co^2+^, Mn^2+^and Mg^2+^ ions had little effects on the activity, whereas Fe^2+^, Cu^2+^ and Ni^2+^ inhibited the activity at a final concentration of 1 mM. On the other hand, 1 mM of Ca^2+^ or Ba^2+^ activated the cyclization activity of Opt-CGT up to 114.9 and 111.7%, respectively. The effects of metal ions and the metal chelator on the recombinant Opt-CGT and non-optimized CGT were almost similar [14].

**Table 3:** Effects of bivalent metal ions and chemical agents on Opt-CGT activity

Additives	Concentration	Relative activity (%)
Metal ions		
No addition		100 ± 1.8
Fe^2+^(FeCl_2_)	1 mM	80.6 ± 3.5
Mg^2+^(MgCl_2_)	1 mM	96.6 ± 1.9
Cu^2+^(CuCl_2_)	1 mM	59.9 ± 4.8
Ca^2+^(CaCl_2_)	1 mM	114.9 ± 2.4
Ni^2+^(NiCl_2_)	1 mM	79.3 ± 3.9
Zn^2+^(ZnCl_2_)	1 mM	97.3 ± 2.7
Ba^2+^(BaCl_2_)	1 mM	111.7 ± 2.2
Mn^2+^(MnCl_2_)	1 mM	101.8 ± 3.1
Co^2+^(CoCl_2_)	1 mM	94.1 ± 2.3
EDTA	1 mM	92.5 ± 2.8
Organic solvents		
Methanol	10%	36.9 ± 3.2
Ethanol	10%	13.8 ± 1.2
Acetonitrile	10%	5.5 ± 0.9
Isopropanol	10%	18.9 ± 2.8
DMSO	5%	74.2 ± 2.3
Surfactants		
Tween-20	20 mg·mL^−1^	32.4 ± 3.1
Tween-80	20 mg·mL^− 1^	55.8 ± 3.7
Triton X-100	20 mg·mL^−1^	87.1 ± 2.4
SDS	2 mg·mL^−1^	36.9 ± 3.1
Enzyme inhibitors		
Urea	20 mg·mL^−1^	97.6 ± 2.7

The effects of various chemical agents on the enzyme activity were also assessed (Table 3). Methanol, ethanol, acetonitrile, isopropanol, Tween 20, Tween 80 and SDS strongly inhibited Opt-CGT enzyme activity, whereas DMSO and Triton X-100 slightly reduced its activity. Urea had no significant influence on the enzyme activity. These data suggested that Opt-CGT was a metal ion non-dependent enzyme and its organic solvent tolerance is poor.

Overall, the biochemical characteristics are almost the same between Opt-CGT and native CGTase (Table 4). However, the half-life of the native enzyme at 50 °C is 48 min, whereas the Opt-CGT was just about 20 min, indicating that the native CGTase has a better thermal tolerance. Interestingly, there was some improvement in the pH stability of Opt-CGT, suggesting that the Opt-CGT still maintained an excellent stability at the pH 11.0.

**Table 4:** Comparison of the properties between native CGTase and Opt-CGT

Details	Opt-CGT (This study)	Native CGTase
Induction time (h)	20	70
Molecular mass	74 kDa	75 kDa
Optimum temperature (°C)	50	50
Half-life time of the CGTases at 50 °C (min)	~ 20	~ 48
Optimum pH	6.0	5.5
pH stabilities (The activity of CGTases retained > 80%)	6.0–11.5	6.0–10.0

### Production of AA-2G

**Fig. 4:**
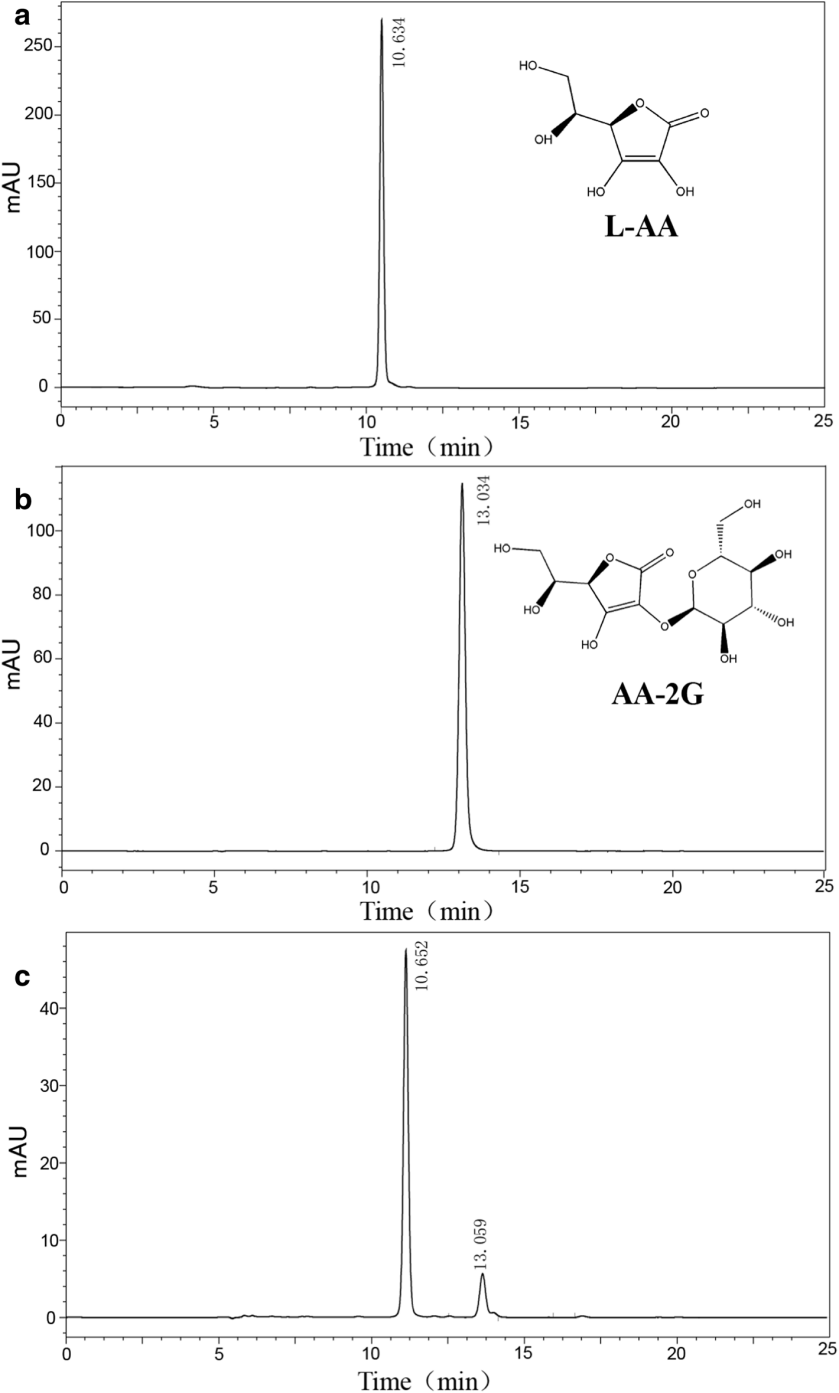
HPLC analysis of AA-2G production by the Opt-CGT. **a** the standard sample of L-AA (0.1 g/L); **b** the standard sample of AA-2G (0.1 g/L); **c** reaction products obtained: The peak I is the rest of L-AA and the peak II is the generated AA-2G. The reaction sample was diluted 1000 times before analysis

Many oligosaccharides and polysaccharides could be used as substrates for the transformation of AA-2G. Usually, α-CD was the ideal glycosyl donor for AA-2G production. The highest concentration of AA-2G reached 9.1 g/L when the temperature, pH, amount of enzyme, concentration of L-AA and α-CD were 40 °C, 5.0, 60 U, 100 g/L and 100 g/L, respectively (Fig. 4). Jun et al. reported that AA-2G production concentration was 1.53 g/L using 30 g/L of AA, 70 g/L of glycosyl donor and 2000 U/mL of CGTase in the catalytic system, which was much lower than this study [4].

In addition, due to the relatively high price of α-CD, a lot of other glycosyl donors were selected, such as β-CD, soluble, corn and cassava starch. When β-cyclodextrin was used as the glycosyl donor, another cyclodextrin composed of seven glucose molecules, 4.9 g/L of AA-2G could be obtained, and the decreased production maybe because the lower solubility of β-cyclodextrin. However, the cost of β-cyclodextrin is still expensive for industrial production. To further decrease the cost, starches were thought to act as the glycosyl donors, and the production of AA-2G reached 3.5 , 1.3 and 1.5 g/L from soluble, corn and cassava starch, respectively. Compared to the price of different starches, corn and cassava starch are more economic than soluble starch. However, when different starches were used as the glycosyl donors, the production of AA-2G was still at a relatively low level. Hence, future study should be performed to improve the AA-2G production from economic glycosyl donor.

## Conclusions

The α-CGTase encoding gene from *P. macerans* JFB05–01 was successfully codon-optimized and expressed intracellularly in *E. coli* BL21 (DE3) . The effects of N-terminal amino acids on activity of CGTase were also confirmed by truncation experiment. Then the recombinant enzyme was purified with a 55% yield by Ni^2+^-NTA resin and characterized its biochemical properties. In order to improve the production of AA-2G, the catalytic conditions are optimized based on the characterizations of purified Opt-CGT. In addition, considering the relatively high cost of cyclodextrin, the glycosyl donor could be changed to starches, but the production should be further improved.
